# Low back pain patients in Sweden, Denmark and the UK share similar characteristics and outcomes: a cross-national comparison of prospective cohort studies

**DOI:** 10.1186/s12891-015-0824-7

**Published:** 2015-11-26

**Authors:** Alice Kongsted, Laura Davies, Iben Axen

**Affiliations:** The Nordic Institute for Chiropractic and Clinical Biomechanics, Odense, Denmark; Departments of Sports Science and Clinical Biomechanics, University of Southern Denmark, Campusvej 55, 5230 Odense, M Denmark; Anglo-European College of Chiropractic, Bournemouth, UK; Institutet för Miljömedicin, Karolinska Institutet, Stockholm, Sweden; Department of Regional Health Research, University of Southern Denmark, Odense, Denmark

**Keywords:** Chiropractic, Cross-national comparison, Longitudinal studies, Low back pain, Musculoskeletal pain, Primary health care, Prognosis

## Abstract

**Background:**

Low back pain (LBP) is the world’s leading cause of disability and yet poorly understood. Cross-national comparisons may motivate hypotheses about outcomes being condition-specific or related to cultural differences and can inform whether observations from one country may be generalised to another. This analysis of data from three cohort studies explored whether characteristics and outcomes differed between LBP patients visiting chiropractors in Sweden, Denmark and the UK.

**Methods:**

LBP patients completed a baseline questionnaire and were followed up after 3, 5, 12 and 26 weeks. Outcomes were LBP intensity (0–10 scales) and LBP frequency (0–7 days the previous week). Cohort differences were tested in mixed models accounting for repeated measures. It was investigated if any differences were explained by different baseline characteristics, and interaction terms between baseline factors and nations tested if strength of prognostic factors differed across countries.

**Results:**

The study sample consisted of 262, 947 and 453 patients from Sweden, Denmark and the UK respectively. Patient characteristics were largely similar across cohorts although some statistically significant differences were observed. The clinical course followed almost identical patterns across nations and small observed differences were not present after adjusting for baseline factors. The associations of LBP intensity and episode duration with outcome differed in strength between countries.

**Conclusions:**

Chiropractic patients with low back pain had similar characteristics and clinical course across three Northern European countries. It is unlikely that culture have substantially different impacts on the course of LBP in these countries and the results support knowledge transfer between the investigated countries.

## Background

Low back pain (LBP) was in 2010 estimated to be the condition with the highest impact on global health in terms of years lived with disability [1], and there is great motivation for reducing the burden of LBP both from an individual and a public health perspective. However, LBP is a highly heterogeneous condition and it is poorly understood why it is a severe and disabling condition in some individuals while being rather inconsequential in others [2–4].

Prognostic estimates differ quite substantially across cohorts [5, 6]. The reasons for such differences are not fully understood and may be due to variation in outcome measures or to study bias. However, differences may also be caused by varied representations of people with a good or poor prognosis, or there may be underlying differences across settings, countries or cultures explaining these observations. A systematic review illustrated that the prognosis of LBP reported in Australian studies was more favourable than those reported from Europe and the United States [6], but outcome measures differed and direct comparisons of the prognosis of LBP across countries are, to the best of our knowledge, non-existent.

Exploring similarities and differences between settings and nations are of interest for at least two reasons: first, it reveals whether observations from one country and culture may be generalised to another. Second, it may motivate hypotheses about outcomes being condition-specific or due to differences between settings. Furthermore, recognition of substantial similarities may support the design of multicentre or cross-national research projects. Large-scale studies are of special interest in an extremely common and costly condition like LBP where even small treatment effects may be worthwhile. It takes large sample sizes to demonstrate that such effects are consistent, and it also takes large trials to investigate if certain subgroups of patients may achieve larger treatment effects than others [7].

Comparing the prognosis of LBP across cohorts is complicated by the fact that LBP is an episodic or fluctuating condition [8]. To date neither an obvious definition of recovery nor an optimal time-point for follow-up has been offered. Frequently repeated measures of outcome over a fairly long time may best describe the course of LBP and allow comparison of any cohort differences.

In this study, we used existing datasets containing frequent follow-ups of subjects from three Northern European countries: Sweden (SE), Denmark (DK) and the United Kingdom (UK). The patients were all seeking care for LBP and the setting was similar: chiropractic practices. Thus, we were able to investigate if LBP cohorts from settings and countries that in many aspects were expected to be comparable actually showed similar characteristics and clinical courses of LBP. Additional purposes were to explore to what extent any observed differences in outcome could be explained by measured baseline characteristics, and to investigate if any of the baseline factors had different prognostic strength across these countries.

## Method

This explorative study was a post-hoc analysis of data collected in three separately conducted longitudinal cohort studies based in chiropractic practices in SE [9], DK [10, 11] and the UK (unpublished). The Swedish data collection was approved by the local ethics committee at the Karolinska Institutet (2007/1458-31/4) and the UK study by the Anglo-European College of Chiropractic Ethics Sub-Committee (notified by letter dated 28th August 2008). The Regional Ethics Committee for Southern Denmark was advised about the Danish data collection, but according to Danish law, a study that does not contain invasive tests or interventions aimed at individuals does not require ethics approval [12]. Written consent of participation was obtained from all participants.

### The chiropractic settings

Chiropractors in the three participating countries are authorised as primary health care providers for the diagnosis, treatment and prevention of musculoskeletal problems. Patients can seek care without a referral and most costs are covered by self-payment.

In SE, the data collection was conducted by a convenience sample of 35 chiropractors. The DK data collection was conducted by chiropractors in 17 clinics that are affiliated with the Nordic Institute of Chiropractic and Clinical Biomechanics as research clinics. The UK data were collected by a convenience sample of 65 chiropractors, all of whom were practising members of the British Chiropractic Association. In the UK, although the majority of chiropractors work in private practice, some do provide services through the National Health Service, where all costs are covered.

The chiropractors in these three North European countries share some common features, mainly related to the fact that they are members of professional associations that ensure academic standards and continued professional development. A majority of the SE chiropractors were trained in the UK (as were the UK chiropractors), while the majority of the DK clinicians were trained in Denmark [13].

### Participants

Participating chiropractors were instructed to invite consecutive patients, who were seeking care for LBP with or without leg pain, to the study. The subjects were aged 18–65 years (18–60 years in the UK study) and had not been under chiropractic care for at least three months. Patients were not included if pregnant, if unable to understand and read the native language, or if they were not able to respond to a text message via a mobile phone.

### Baseline measures

In SE, patients were included at the second visit to the chiropractor, while in DK and the UK, patients were included at the first consultation. Patient-completed questionnaires included information regarding: age, sex, any previous LBP episodes (Yes/No), many previous LBP episodes (Yes/No), LBP days last year (≤30 days/>30 days), any sick leave due to LBP (Yes/No), LBP intensity (0–10 numeric rating scale), leg pain (Yes/No). Differences in variables across cohorts are summarised in Table 1.

**Table 1:** Overview of definitions of those variables that were defined differently across three cohorts of chiropractic patients

	Sweden	Denmark	UK
Any previous LBP episodes	not available	Yes: ≥1 previous episode ever	Yes: ≥1 previous episode ever
		No	No
≥3 previous LBP episodes	Yes: ≥ 4 episodes previous year	Yes: ≥3 previous episodes ever	not available
	No: < 4 episodes previous year	No: <3 previous episodes ever	
LBP days last year	≤30: 30 days or less in total last year	≤30: 30 days or less in total last year	≤30: Max 30 days on and off last year *or* Max 30 days constant last year
	>30: More than 30 days in total last year, intermittent pain *or* More than 30 days in total last year, daily pain	>30: More than 30 days in total last year	>30: More than 30 days on and off last year *or* More than 30 days constant last year
Any sick leave due to LBP	Yes: ≥1 day within the last year	Yes: ≥1 day within the last month	Yes: ≥1 day of current sick leave
	No: No sick leave within the last year	No: No sick leave within the last month	No: No current sick leave
Leg pain	Yes	Yes: Leg pain intensity last 24 h = 1–10	Yes: Yes, leg pain above knee *or* Yes, leg pain below knee
	No	No: Leg pain intensity last 24 h = 0	No: No leg pain

### Outcome measures

Follow-ups were conducted using SMS-track, which is an automated system sending text messages with follow-up questions to participants [14]. The response is given by answering the SMS with a number which is stored directly in a database.

In SE, participants were asked weekly for 26 weeks about the number of days with bothersome LBP the previous week (hereafter referred to as *bothersome LBP days*) and could respond with a number from zero to seven.

In DK, participants were asked weekly for 52 weeks about number of days with LBP the previous week (hereafter referred to as *LBP days*) with response options as in SE. If reporting any *LBP days* they were also asked about LBP intensity on a 0–10 scale (referred to as *LBP intensity*). If zero *LBP days* were reported, *LBP intensity* was defined as zero.

In the UK, participants were asked about *LBP intensity* on a 0–10 scale daily over one week following the 1st visit to the chiropractor. At the 4th visit and after 3 and 6 months, the same question was asked in a paper questionnaire.

### Data analyses

Data were cleaned (detection and removal of inappropriate answer options) and prepared for the original purposes in the respective research units and merged in STATA 13.1 (StataCorp, Texas, USA) which was used for all analyses.

Missing values on baseline variables were imputed by multiple imputations based on fully conditional specifications with five chained iterations. Using the same method, missing values in the daily measures of pain during the first week in the UK data were imputed and a sum score of these were calculated if a minimum of four out of seven were available to represent a measure of *LBP days* in week one. Missing values on other follow-up measures were not imputed.

The mean of seven daily scores on LBP intensity collected over one week in the UK represented *LBP intensity* in week one. The timing of the 4th visit in the UK was registered as 2–4, 4–6, 6–8 weeks and more than 8 weeks after the first visit. To align time-points with data from DK and SE, the first two mentioned time points were included in the analysis as 3-week and 5-week follow-ups respectively. Less than 5 % of cases had the 4^th^ visit later than this.

Baseline characteristics were described as proportions with 95 % confidence intervals (95 % CI) and medians with interquartile ranges (IQR). Differences across nations were tested by means of chi-squared, ANOVA (normally distributed continuous measures) or Kruskall Wallis test (non-normally distributed continuous measures).

Comparisons of the course of LBP between the cohorts were made in three steps: (1) comparing the course of LBP based on the outcome *LBP days* to that based upon *LBP intensity* in the DK cohort to explore to what extent the observed course was affected by the outcome measure. In a previous study, these two measures provided very similar descriptions of the clinical course of LBP [15]. Then (2) comparing *bothersome LBP days* and *LBP days* between SE and DK and (3) comparing *LBP intensity* between DK and the UK. Comparisons were illustrated in time series plots and differences were tested for statistical significance at time points 3, 5, 12, and 26 weeks that were available from all cohorts. Nation differences were tested in mixed models with *LBP days* / *LBP intensity* as outcome and country, time (categorical), and the interaction between country and time as explanatory variables. Individuals and clinics were introduced as random effects to account for correlation between time measures and clustering effects within clinics.

To investigate if any observed differences could be explained by measured baseline variables, these variables and their interactions with country were introduced as covariates in an adjusted model. Non-significant interactions (p > .1) were removed from the models. Significant interactions between a prognostic factor and country in these models were interpreted as this factor having different associations with outcome in the countries. This was merely explorative since no pre-hoc hypotheses were established about possible national differences and the selection of covariates was not theoretically founded.

## Results

### Participants

**Fig. 1:**
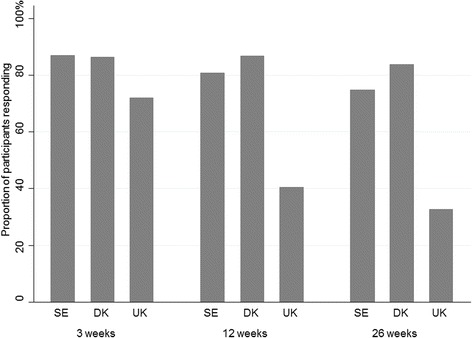
Response rates in three cohorts at three follow-up time points. The response rate in the UK in week 3 is the proportion participating at the 4th visit no matter when that visit was. DK: Denmark; SE: Sweden; UK: The United Kingdom

The study sample consisted of 262, 947 and 453 patients from SE, DK and the UK respectively. Follow-up rates are illustrated in Fig. 1 The combined response rates of the three cohorts after 3 weeks (or at the 4th visit in the UK), 12 weeks and 26 weeks were 82 %, 73 % and 68 % with rather similar response rates in SE and DK and a smaller completion rate (60 % and 77 % at 12 and 26 weeks, respectively) from the UK study. There was no difference between participants and those who did not complete the last follow-up regarding sex, number of LBP days last year, sick leave, episode duration, leg pain or LBP intensity (results not shown). In the UK sample, participants who dropped out were on average 5.5 years younger than those completing the 26-week follow-up.

### Baseline characteristics

Baseline characteristics of participants are summarised in Table 2. Statistically significant differences were observed between countries on most measured characteristics but most were small or modest in size. As compared to DK and the UK, the SE patients reported more often >30 days with LBP the last year, lower LBP intensities, and less sick leave. More DK than UK patients had very short duration of LBP (0–2 weeks). The prevalence of leg pain varied from 45 % in UK, over 51 % in SE to 58 % in DK.

**Table 2:** Patient reported baseline characteristics of chiropractic patients in three European countries

	Sweden, n = 262	Denmark, n = 947	UK, n = 453
Females, % (95 % CI)	48 % (42 %–54 %)	45 % (42 %–48 %)	47 % (42 %–51 %)
Age years*, median (IQR)	44 (35–52)	43 (34–53)	41 (34–49)
Any previous LBP episodes*, % (95 % CI)	NA	84 % (81 %–86 %)	88 % (85 %–91 %)
≥3 previous LBP episodes*, % (95 % CI)	48 % (42 %–54 %)	49 % (45 %–52 %)	NA
More than 30 LBP days last year*, % (95 % CI)	56 % (50 %–62 %)	25 % (22 %–28 %)	37 % (32 %–41 %)
Sick leave#, % (95 % CI)	18 % (14 %–24 %)	22 % (20 %–25 %)	27 % (23 %–31 %)
	within last year	within last month	current sick leave
Episode duration*, % (95 % CI)	NA		
0-2 weeks		62 % (59 %–66 %)	49 % (45 %–54 %)
2 weeks–3 months		24 % (21 %–27 %)	34 % (30 %–39 %)
More than 3 months		13 % (11 %–16 %)	17 % (14 %–21 %)
LBP intensity (0–10)*, median (IQR)	4 (3–6)	7 (5–8)	6 (5–8)
Leg pain*, % (95 % CI)	51 % (45 %–57 %)	58 % (55 %–61 %)	45 % (40 %–49 %)

*Significant association with country (*p* < .05)

^#^Because of different timing differences were not tested for statistical significance

*CI* confidence interval, *IQR* interquartile range, *NA* not available

### Comparing LBP days and LBP intensity in the Danish cohort

**Fig. 2:**
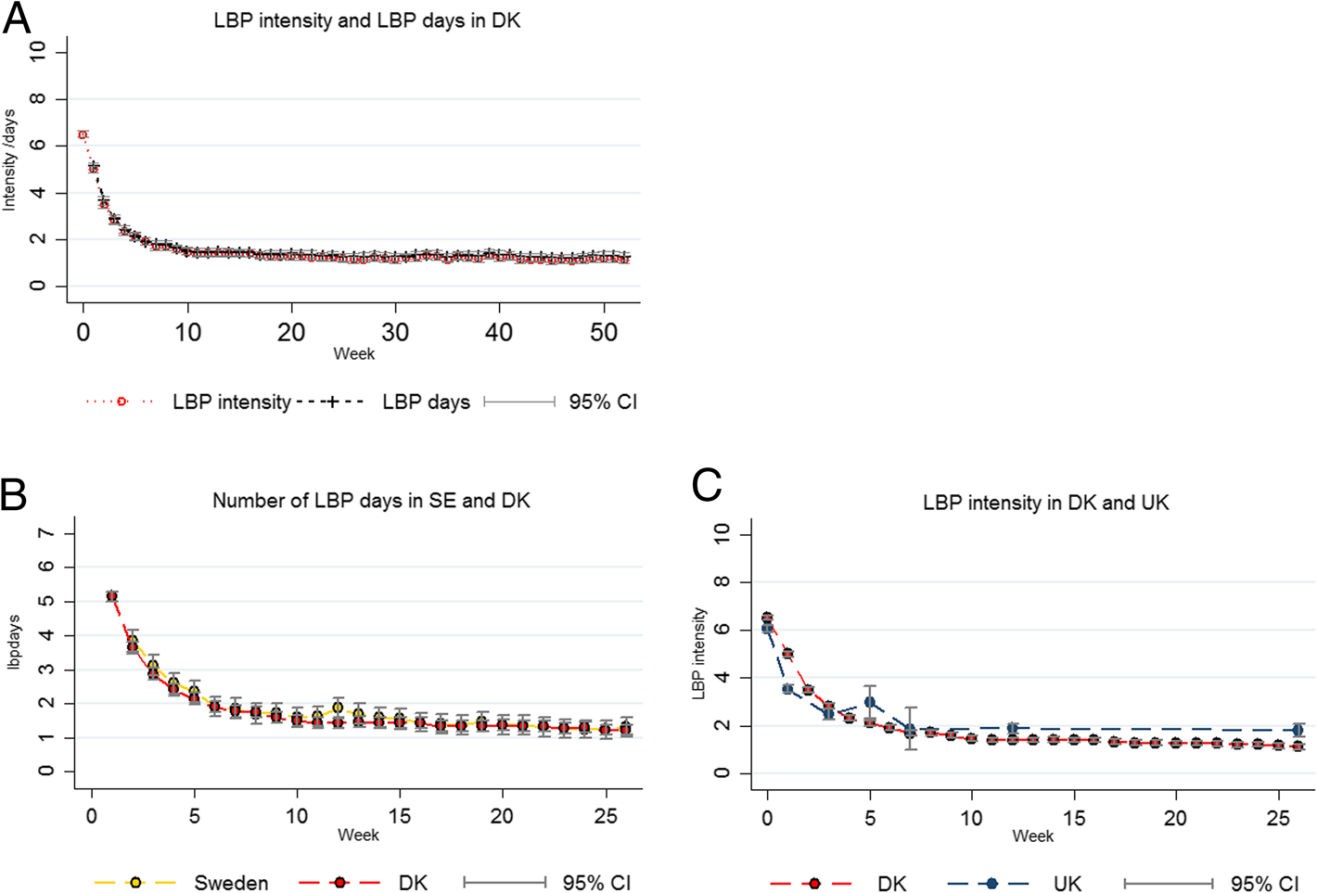
Observed LBP outcomes in cohorts from Sweden (SE), Denmark (DK) and the UK. **a** Mean LBP intensity and mean number of LBP days in DK. **b** Mean number of LBP days in DK and SE. **c** Mean LBP intensity in DK and the UK

The course of population averaged *LBP days* and *LBP intensity* followed identical patterns in the DK cohort (Fig. 2). The scores on *LBP days* across weeks 1, 3, was on average .08 (95 % CI .01-.16) point higher than the scores on *LBP Intensity* measured on the two original scales.

### Comparing SE and DK

The trajectories of *bothersome LBP days* and *LBP days* were very similar in the SE and DK cohorts (Fig. 2). Differences between countries were small and not statistically significant (.2 (95 % CI:−.5 to .04)). The individual variation on *LBP days* was large and similar in the two countries (Standard deviations (sd) across all time points: SE sd = 2.4; DK sd = 2.5).

After adjusting for baseline variables there were still no country differences. The only covariate that had a significant interaction with country was LBP intensity, suggesting that higher baseline LBP intensity was less influential in DK than in SE (β-coefficients for pain on 0–10 NRS scales were .19 (95 % CI .09-.30) and .07 (95 % CI .01-.13) for SE and DK respectively).

### Comparing DK and the UK

*LBP intensity* followed similar general patterns in the DK and UK cohorts (Fig. 2). However, a smaller reduction in *LBP intensity* was observed after the third week in the UK than DK, and *LBP intensities* were slightly higher in the UK cohort at the 5-weeks (1.1 point; 95 % CI [.4–1.8]), the 12-weeks (.5 point; 95 % CI [.2–.8]), and the 26-weeks (.7 point; 95 % CI [.3–1.1]) follow-ups. There was considerable individual variation within in cohorts of similar magnitude (DK sd=2.9; UK sd = 3.0).

After adjusting for baseline characteristics, the reduction in *LBP intensity* was still steeper in DK than the UK (Table 3). However, estimated *LBP intensities* only differed significantly after 3 weeks with 1.2 points (95 % CI .6; 1.8) lower pain in the UK than in DK. After 5 weeks, 12 weeks and 26 weeks, adjusted country differences were non-significant implying that the crude differences observed at these time points could be explained by differences in measured baseline factors. In addition, country had significant interactions with baseline LBP intensity and episode duration suggesting that higher pain intensity had a stronger association with poor outcome in the UK than in DK, whereas episode duration was less influential in the UK than in DK (Table 3).

**Table 3:** Mixed linear models analysis comparing the outcome of *LBP intensity* (measured on a scale from 0 to 10) between the DK and the UK cohorts

	Beta-coefficient	*p*-value	Beta-coefficient	*p*-value
	unadjusted model		adjusted model*	
Baseline *LBP intensity* (ref.: DK)				
UK	-.5 (−.7;−.2)	<.001	−1.4 (−1.9;−.8)	<.001
Week (ref.: week 0)		<.001		<.001
3	−3.7 (−3.9;−3.5)		−3.7 (−3.9;−3.6)	
5	−4.4 (−4.6;−4.2)		−4.4 (−4.6;−4.2)	
12	−5.1 (−5.3;−5.0)		−5.1 (−5.3;−5.0)	
26	−5.4 (−5.6;−5.2)		−5.4 (−5.6;−5.2)	
Country#Week (ref.: DK week 1)		<.001		<.001
UK week 3	.1 (−.3; .6)		.2 (−.2; .6)	
UK week 5	1.6 (.9; 2.3)		2.1 (1.4; 2.7)	
UK week 12	1.0 (.6; 1.3)		1.0 (.6; 1.3)	
UK week 26	1.2 (.8; 1.6)		1.2 (.8; 1.6)	
LBP (0–10) (ref: DK)			.3 (.3; .4)	<.001
LBP#UK			.2 (.1; .3)	<.001
Episode duration (ref. <2 weeks)				<.001
2 weeks–3 months			.5 (.3; .7)	
More than 3 months			.8 (.6; 1.1)	
Country#Duration (ref. DK <2 weeks)				
UK 2 weeks–3 months			-.5 (−.8; −.1)	<.01
UK More than 3 months			-.5 (−.9;-.03)	

*:The model included all baseline variables as covariates. Only significant (*p* < .05) interactions are presented

#:Interaction term. The interaction defines the difference in the beta coefficients between countries. For example the coefficient for LBP was 0.3 in DK and 0.3 + 0.2 = 0.5 in UK

## Discussion

This study presented the first direct comparison of LBP outcomes across nations by merging three existing data sets. It was demonstrated that patients seeking care from chiropractors in three Northern European countries had generally similar clinical characteristics and course of symptoms. The course of LBP was characterised by substantial improvement during approximately five weeks after seeking care, and practically no improvement after ten weeks when considering cohort averages. The SE cohort included patients with more previous LBP days and less intense pain than was observed in the other cohorts, whereas many DK patients had LBP of very short duration. No differences in the course of LBP were detected comparing SE and DK, and the small differences observed between DK and the UK could mostly be explained by cohort differences at inclusion. In all cohorts there was considerable individual variation on outcomes indicating that the cohorts included a heterogenic mixture of patients. The identification of individual course profiles has been addressed elsewhere [9, 16].

Most associations between baseline factors and outcome did not differ significantly between countries, which implies that factors such as previous LBP history, leg pain and sick leave are prognostic factors that were equally important in these cohorts. Although these prognostic factors have been identified in other settings [3, 17], a direct comparison of their strength of association across countries has not previously been available. Baseline LBP intensity had a stronger association with outcome in SE and the UK than in DK, and it could be hypothesised that high pain intensity is less influential when present at the first consultation than if still reported at the second visit when baseline registrations in SE were made. Moreover, longer episode duration was more strongly associated with poorer outcome in DK than in the UK. As patients in the UK presented with longer episode duration than DK patients this may suggest that care seeking is triggered by different factors in these two countries or the patients may differ in their understanding of ‘present episode’.

The obvious strength of the present study was the availability of three relatively large cohorts all included from the same type of care setting, defined by the same inclusion criteria and with some comparable baseline variables. Moreover, all studies involved repeated measurements of outcomes. On the other hand, the study was limited by being a secondary analysis which meant that slightly different definitions of variables were used and also that the three countries could be compared only in pairs due to different outcome measures. Still, we believe this indirect comparison of all three countries was reasonable since both of the investigated outcomes, *LBP intensity* and *LBP days*, were available from one cohort and were shown to have identical trajectories. A further uncertainty results from baseline registrations being performed at the second visit in SE which might explain the lower mean LBP intensity at inclusion in this cohort as compared to the UK and DK.

Response rates were high in the SE and DK studies, but lower in the UK study. It is not known to what extent drop-outs may have influenced the cohorts differently. However, the only observed difference between responders and non-responders in the UK was that the latter were younger, and age was not associated with outcome in our models.

Data concerning content and frequency of treatment were not collected and the actual treatment could not be controlled for. However, as the majority of the UK and SE chiropractors share the same educational background and the differences in outcome were similar across countries, the influence of variability in treatment on the outcome was probably minor. It is expected that the majority of patients received advice and spinal manipulative therapy often combined with exercises and soft-tissue techniques [18, 19].

It should be noted that patients are mostly self-referred to chiropractic care and this self-selection limits the generalisability to other primary care patient populations, such as physiotherapy practice (where patients are generally referred) and general practice (which does not involve self-payment in the investigated countries). In DK, chiropractic patients have been shown to differ substantially from LBP patients consulting a general practitioner [20], but such data are not available for SE and the UK. It would be interesting to conduct a similar cross-country comparison of baseline and outcome variables in patients from other primary health care settings, to investigate the possibility of pooling data.

## Conclusions

This cross-national comparison demonstrated that chiropractic patients with LBP had similar characteristics and clinical course across three Northern European countries. Thus, it is unlikely that factors such as cultural perceptions of pain or health care systems have substantially different impacts on the course of LBP in chiropractic cohorts in these countries. Therefore, these results support knowledge transfer across the investigated countries. However, earlier care seeking in DK as compared to the UK and long episode duration being more strongly related to a poor prognosis in DK raise questions about potential differences in what triggers care seeking. Based on our results it seems reasonable to coordinate collaborative data collections across these countries when research questions demand high sample sizes. When interpreting these results, it should be kept in mind that chiropractic is a distinct part of primary health care and more pronounced differences between the investigated countries may exist between different types of primary health care.

## Abbreviations

DK: Denmark
LBP: low back pain
SE: Sweden
UK: United Kingdom

## References

[1] Vos T, Flaxman AD, Naghavi M, Lozano R, Michaud C, Ezzati M (2012). Years lived with disability (YLDs) for 1160 sequelae of 289 diseases and injuries 1990–2010: a systematic analysis for the Global Burden of Disease Study 2010. Lancet.

[2] Hayden JA, Dunn KM, van der Windt DA, Shaw WS (2010). What is the prognosis of back pain?. Best Pract Res Clin Rheumatol.

[3] Kent PM, Keating JL (2008). Can we predict poor recovery from recent-onset nonspecific low back pain? A systematic review. Man Ther.

[4] Verkerk K, Luijsterburg PA, Miedema HS, Pool-Goudzwaard A, Koes BW (2012). Prognostic factors for recovery in chronic nonspecific low back pain: a systematic review. Phys Ther.

[5] Da C, Menezes Costa L, Maher CG, Hancock MJ, McAuley JH, Herbert RD (2012). The prognosis of acute and persistent low-back pain: a meta-analysis. CMAJ.

[6] Itz CJ, Geurts JW, Van Kleef M, Nelemans P (2013). Clinical course of non-specific low back pain: a systematic review of prospective cohort studies set in primary care. Eur J Pain.

[7] Brookes ST, Whitely E, Egger M, Smith GD, Mulheran PA, Peters TJ (2004). Subgroup analyses in randomized trials: risks of subgroup-specific analyses; power and sample size for the interaction test. J Clin Epidemiol.

[8] Axen I, Leboeuf-Yde C (2013). Trajectories of low back pain. Best Pract Res Clin Rheumatol.

[9] Axen I, Bodin L, Bergstrom G, Halasz L, Lange F, Lovgren PW (2011). Clustering patients on the basis of their individual course of low back pain over a six month period. BMC Musculoskelet Disord.

[10] Kongsted A, Vach W, Axo M, Bech RN, Hestbaek L (2014). Expectation of recovery from low back pain: a longitudinal cohort study investigating patient characteristics related to expectations and the association between expectations and 3-month outcome. Spine (Phila Pa 1976).

[11] Eirikstoft H, Kongsted A (2014). Patient characteristics in low back pain subgroups based on an existing classification system. A descriptive cohort study in chiropractic practice. Man Ther.

[12] Danish National Commitee on Biomedical Research Ethics. Guidelines about Notification. http://www.cvk.sum.dk/English/guidelinesaboutnotification.aspx

[13] Lemeunier N, Kongsted A, Axen I (2011). Prevalence of pain-free weeks in chiropractic subjects with low back pain-a longitudinal study using data gathered with text messages. Chiropr Man Ther.

[14] Intelligent Communication with SMS-Track [http://www.sms-track.com]

[15] Kongsted A, Leboeuf-Yde C (2010). The Nordic back pain subpopulation program: course patterns established through weekly follow-ups in patients treated for low back pain. Chiropr Osteopat.

[16] Kongsted A, Kent P, Hestbaek L, Vach W (2015). Patients with low back pain had distinct clinical course patterns that were typically neither complete recovery nor constant pain. A Latent Class Analysis of longitudinal data. Spine J.

[17] Grotle M, Foster NE, Dunn KM, Croft P (2010). Are prognostic indicators for poor outcome different for acute and chronic low back pain consulters in primary care?. Pain.

[18] Nielsen OL, Kongsted A, Christensen HW (2015). The Chiropractic Profession in Denmark 2010–2014. A descriptive report.

[19] Axen I, Rosenbaum A, Robech R, Wren T, Leboeuf-Yde C (2002). Can patient reactions to the first chiropractic treatment predict early favorable treatment outcome in persistent low back pain?. J Manipulative Physiol Ther.

[20] Hestbaek L, Munck A, Hartvigsen L, Jarbol DE, Sondergaard J, Kongsted A (2014). Low back pain in primary care: a description of 1250 patients with low back pain in danish general and chiropractic practice. Int J Family Med.

